# A Hybrid Multi-Criteria Approach to the Vendor Selection Problem for Sensor-Based Medical Devices

**DOI:** 10.3390/s23020764

**Published:** 2023-01-09

**Authors:** Constanta Zoie Radulescu, Marius Radulescu

**Affiliations:** 1 National Institute for Research and Development in Informatics, 8-10, Mareşal Averescu, 011455 Bucharest, Romania; 2 Gheorghe Mihoc-Caius Iacob Institute of Mathematical Statistics and Applied Mathematics, Romanian Academy, Calea 13 Septembrie, No.13, 050711 Bucharest, Romania

**Keywords:** sensor-based medical devices, medical sensors, vendor selection, multi-objective model, flexible best worst method, criteria weights, performance, costs

## Abstract

Sensors for health are a dynamic technology and sensor-based medical devices (SMD) are becoming an important part of health monitoring systems in healthcare centers and ambulatory care. The rapid growth in the number, diversity and costs of medical devices and Internet of Things (IoT) healthcare platforms imposes a challenge for healthcare managers: making a rational choice of SMD vendor from a set of potential SMD vendors. The aim of this paper is to develop a hybrid approach that combines a performance evaluation model and a multi-objective model for the SMD vendor selection problem. For determining the criteria weights in the performance evaluation model, an original version of the best worst method (BWM) is applied, which we call the flexible best worst method (FBWM). The multi-objective model has two objective functions; one is to maximize the SMD performance and the other is to minimize the SMD cost. A case study for the application of the hybrid approach for SMD procurement in a healthcare center is analyzed. The hybrid approach can support healthcare decision makers in their SMD procurement decisions.

## 1. Introduction

Cloud computing, the Internet of Things (IoT), artificial intelligence and extender reality are used to monitor patients and deliver new treatments and services. Now, integrated medical technologies and information technologies revolutionize the way in which hospitals, healthcare centers, and care providers communicate with each other and with their patients. IoT is an emerging technology that is enabling communication and the exchange of data and information between devices and systems. IoT is commonly defined as an infrastructure network that connects various sensors or smart objects and allows more data interoperability, device management, communication, and information sharing for application purposes [1].

Sensors for health are a dynamic technology and sensor-based medical devices (SMD) are becoming an important part of health monitoring systems in healthcare centers and ambulatory care. SMD offer a number of new opportunities for healthcare professionals to monitor patients, as well as for patients to monitor themselves. IoT in healthcare basically includes sensors, SMD, software, information processing systems and platforms. The use of the Internet of Things (IoT) with SMD enables easy access to information (e.g., a patient’s vital parameters). The information is transmitted by SMD to IoT where they are stored, aggregated and analyzed. In this paper, we understand SMD devices as medical devices that provide information to an IoT platform.

The rapid growth in the number, diversity and cost of medical devices and IoT healthcare platforms imposes a challenge for healthcare managers: making a rational choice of vendor from a set of potential vendors.

The selection of a vendor in the healthcare industry is a multi-criteria decision-making problem. Procurement managers define the set of vendors, brands, the set of products to be purchased and the set of selection criteria. Quality, price and lead time are common selection criteria. Evaluation of the performance of vendors and selecting the one with the highest quality offer are important tasks.

The decision process for SMD procurement is a complex one. Complexity may arise for various reasons that include uncertainty of information, evaluation, information from different sources, method or combination of methods selection and the weights assigned.

The aim of this paper is to develop a hybrid approach that combines a performance evaluation model and a multi-objective model for the SMD vendor selection problem. For determining the criteria weights in the performance evaluation model an original version of the best worst method (BWM) is applied, which we call the flexible best worst method (FBWM). The multi-objective model has two objective functions; one to maximize the SMD performance and another to minimize the SMD cost. A case study for the application of the hybrid approach for SMD procurement is presented. The hybrid approach can support managers in SMD procurement decisions in healthcare. The hybrid approach has four stages: (a) the definition of the decision problem; (b) computation of criteria weights for SMD, brands and vendors; (c) performance evaluation of SMD, brands and vendors; (d) solving a multi-objective optimization model and finding the optimal vendor, brand and number of products of the same type to be purchased.

The emphasis in our study was placed on sensor-based medical devices because these devices can be integrated into an IoT-type system. The main feature of these types of devices is the possibility to wirelessly transmit information about health parameters to an IoT-type system. An important criterion from the case study refers to communication capacity with an IoT platform. In this paper, SMD are considered as devices that can transmit information wirelessly to an IoT platform.

The subject of our paper was motivated by the complexity of the SMD procurement decision process in healthcare. This domain handles a great diversity of medical devices to be purchased from brands and vendors, which have an impact on the costs and health risks for patients in many ways. Through health applications, the delivery of sensors and medical devices in healthcare can be improved.

The main contributions of the paper are:

- A survey of medical devices and medical sensors;
- A hybrid approach proposal that combines a performance evaluation model and a multi-objective optimization model for SMD, vendor and brand selection;
- Introduction of an original version of the best worst method (BWM), namely, the flexible best worst method (FBWM). The advantages of the FBWM are:
- The number of input data (decision-maker evaluations) is greatly reduced;
- The resulting set of evaluations is fully consistent. As a result, possible reevaluations by the decision-maker are no longer necessary;
- Formulation of a multi-objective optimization model for SMD, vendor and brand selection that maximizes SMD performance and minimizes SMD cost;
- From the above multi-objective optimization model, a single-objective optimization model with a budgetary constraint is formulated. The range of budget parameters is determined.

The structure of the paper is as follows: Section 2 presents a survey of the medical devices and medical sensors. The problem of vendor selection in state-of-the-art devices in the healthcare industry is the subject of Section 3. Section 4 presents FBWM, an original version of the BWM, and Section 5 proposes a hybrid approach that combines a performance evaluation model and a multi-objective optimization model for SMD, vendor and brand selection. In Section 6, a case study that implements the hybrid approach is described. In Section 7, the conclusions of this paper are presented.

## 2. Sensor-Based Medical Devices

IoT medical devices include smartphones, smart sensors, wearable devices, home appliances, medical and industrial instruments. The term IoT has been used as a subject and topic of several studies on medical devices. In the literature, some common terms are used, for example: Internet of Healthcare Things (IoHT) [2,3,4,5], Internet of Medical Things (IoMT) [6,7,8,9] and Ubiquitous healthcare or U-healthcare [10,11,12].

Medical devices based on sensors can detect physical, chemical and biological signals and provide a way for those signals to be measured and recorded. Physical properties that can be sensed include temperature, pressure, vibration, sound level, light intensity, load or weight, flow rate of gases and liquids, amplitude of magnetic and electronic fields, and concentrations of many substances in gaseous, liquid, or solid form. Medical sensors have great advantages in diagnosing, treating and managing diseases, especially for the elderly [13,14,15].

During the COVID-19 pandemic, there has been an increase in telemedicine practices due to established social distancing rules that have led healthcare professionals to consult patients remotely. A smart health monitoring system is being developed using IoT technology which is capable of monitoring the blood pressure, heart rate, oxygen level, and temperature of a person [16]. A survey on COVID-19’s impact on the healthcare domain: worldwide market, implementation, applications, security and privacy issues, challenges and future prospects is presented in [17]. In [18], various applications of IoMT in the context of COVID-19 are presented. This review presents an associated architecture and various other technologies that are deployed to mitigate the virus threat. The paper also highlights the development of new IoMT technologies merged with artificial intelligence (AI), big data, and blockchain.

There are multiple ways in which IoT devices can be used for healthcare. Some of them are: remote patient monitoring, glucose monitoring, heart-rate monitoring, hand hygiene monitoring, depression and mood monitoring, Parkinson’s disease monitoring, connected inhalers, ingestible sensors, connected contact lenses, robotic surgery, etc. IoT device developers, managers and healthcare providers must ensure that they adequately secure data collected by IoT devices.

Remote patient monitoring is the most common application of IoT medical devices. IoT medical devices based on sensors can automatically collect health parameters such as heart rate, blood pressure, temperature, and more from patients who are not physically present in a healthcare institution thus eliminating the need for patients to go to health institutions. When an IoT platform collects patient data, it forwards the data to a software application where healthcare professionals and/or patients can view it. Algorithms may be used to analyze the data in order to recommend treatments or generate alerts.

**Figure 1:**
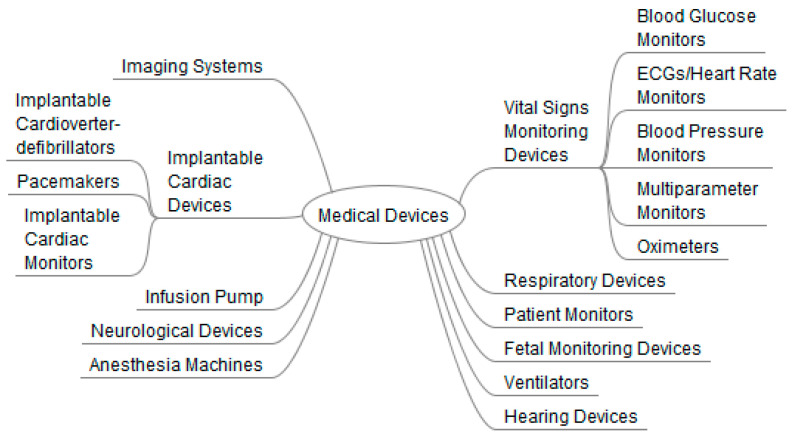
Examples of medical devices.

Medical devices can be divided into the following categories: stationary medical devices, wearable external medical devices, implantable medical devices and other medical devices. Examples of medical devices are presented in Figure 1.

**Figure 2:**
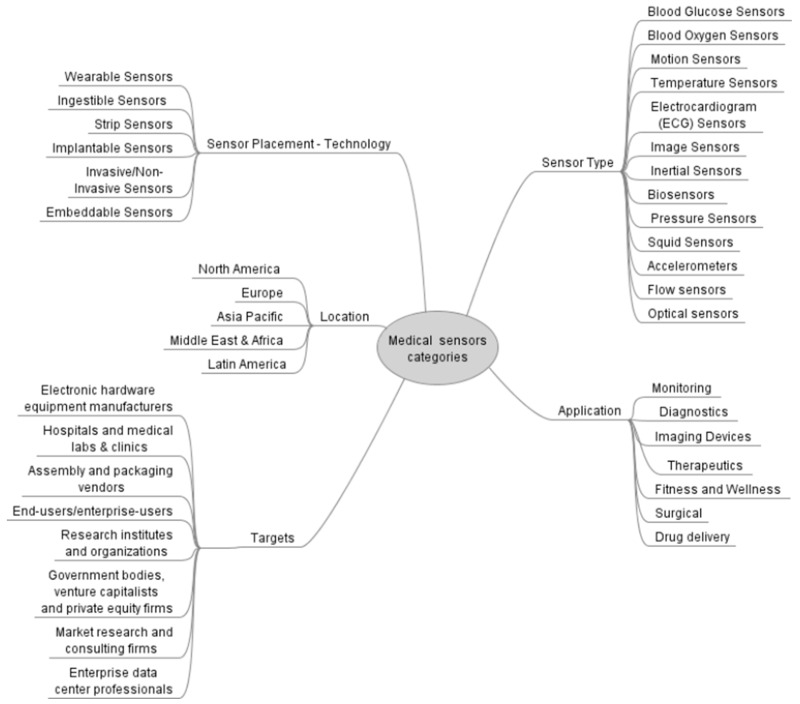
Medical sensor categories.

Medical sensors can be divided in several categories taking into account type of sensor, sensor placement (technology), location, targets and applications (Figure 2).

Much research has been produced in the past years related to the usage of IoT and sensors in healthcare. A review and analysis of popular IoT platforms from different application domains was carried out in [19]. The authors proposed a comprehensive evaluation framework for IoT platforms that considers seven different technical comparison criteria: (1) topology design, (2) programming languages, (3) third-party support, (4) extended protocol support, (5) event handling, (6) security, and (7) privacy. The framework is used to evaluate different IoT platforms highlighting their distinguishing attributes on communications, security, and privacy.

The authors of [20] review scientific articles and patents, for the period 2015–2020, about the Internet of Things (IoT) in healthcare. The aim is to explore both the domain of research and of practice simultaneously by focusing on the most relevant themes concerning IoT application in the healthcare industry.

In [21], a mechanism is proposed for addressing the intersection of the different challenges such as the collection and quality estimation, as well as the interpretation and harmonization of the data that derives from the existing huge amounts of heterogeneous IoT medical devices. A review of the challenges in bio-medical instruments using IoT is also presented in [22].

A review of the literature published from 2007 to 2018 to explore studies in the field of new product development processes, design processes, design methodology, and outcomes of the devices affecting user acceptance is presented in [1].

The authors of [23] provide a summary of the potential healthcare applications of IoT-based technologies. Potential challenges and issues in the IoT health systems are also discussed.

The market of SMD is continuously rising due to an increase in the adoption of medical services at home. With the help of medical equipment that uses sensors, it is possible to monitor patients at home and allow an early diagnosis.

Driving factors of IoT in healthcare have enhanced the growth of the medical device market. Advanced development of the technology of medical devices in the healthcare sector has increased the cost of medical devices in last few years. Advanced software developed in medical devices, advanced sensors and increased research and development of IoT in healthcare has increased the market rate. According to Emergen Research [24], the global IoT medical devices market size was USD 32.63 billion in 2020 and is expected to reach USD 203.13 billion in 2028, registering a robust revenue compound annual growth rate (CAGR) of 25.6% during the forecast period.

## 3. Vendor Selection in Healthcare

According to [25], the healthcare industry is viewed as the world’s largest industry in terms of budget, employees, customers, etc. The market for healthcare products and services has been experiencing explosive growth in recent years due to the increasing demand for medical devices based on sensors and IoT platforms.

Healthcare mangers need to provide high-performance management in order to comply with the growing number of constraints (e.g., budget constraints). This can be done by using efficient decision-making techniques (e. g., optimization methods), instead of inert traditional approaches. An important decision problem in healthcare is the vendor selection problem. Its importance comes from the fact that it has an impact on the performance and the profit of healthcare institutions and from the fact that the health and life of the customers in this sector should be considered. The vendor selection problem is defined as a multi-criteria decision-making (MCDM) problem that includes both quantitative and non-quantitative criteria. The number of papers that study this problem in the healthcare sector is relatively small. In the last decade, applications of vendor selection in the healthcare sector started to grow continuously. Applications of MCDM methods are very popular for solving problems in the healthcare industry.

In the following we present a brief literature review of the literature that concerns vendor selection applications in healthcare institutions.

In [26], a hierarchical structure specific to the healthcare sector was proposed. The criteria within the model were prioritized by considering the evaluations of eight experts from six well-known healthcare organizations in Istanbul. The results of the study show that technical support, terms of payment, and total cost are the three most important criteria in the evaluation process.

The development of an approach for a purchasing portfolio for a large Brazilian hospital is presented in [27]. The approach used the Kraljic model, the fuzzy TOPSIS multi-criteria method and decision rules as methodological resources. The paper classifies the types of devices (called items) and establishes a hierarchy of them but does not provide a solution regarding how many devices can be bought depending on the vendor and brand.

In [28], the problem of strategic procurement of high-cost medical devices is studied. A mixed-integer linear programming (MILP) model is proposed to investigate the use of alternative devices for a set of medical procedures. Model application shows that small changes in pricing parameters and scorecards can have outsized effects on “optimal” shares of business allocated to alternative vendors. This implies the need to revise procurement strategies through time. The authors of the paper do not take into account the weights of the criteria nor the brands in their proposed strategic procurement.

In [29], a holistic MCDM model was developed as a decision support system for the vendor selection in the healthcare sector. Decision-making methods: decision-making trial and evaluation laboratory (DEMATEL) and analytic network process (ANP) were used to analyze the model. The main criteria used in the study were price, quality, logistics, sustainability, and occupational health and safety. The authors of the paper do not take into account the weights of the criteria nor the brands in their proposed strategic procurement.

A vendor selection model for device procurement with application in healthcare was developed in [30]. A decision process for equipment procurement in which a multi-criteria subjective weighting method step-wise weight assessment ratio analysis (SWARA) for equipment evaluation weights and an adaptation of the simple additive weighting (SAW) method for equipment performance are used in combination was proposed.

The authors of [31] had objectives to discover the criteria for the selection of appropriate medical equipment suppliers and to illustrate methods for the selection of a suitable medical equipment vendor. In order to determine the weight of each criterion, the ROC (rank order centroid) method was applied. A fuzzy TOPSIS method was used to select the optimal vendor. A case study was chosen concerning a hospital in the northeast of Thailand [31]. Findings indicated that the main criteria for consideration of vendor selection are quality, price, reliability, agility, compliance, service, benefits and delivery [31].

In [32], the vendor selection problem was addressed with simultaneous consideration of the green and agile indicators for the medical devices industry.

The authors used a hybrid fuzzy decision-making approach based on the fuzzy DEMATEL, fuzzy BWM, fuzzy ANP, and fuzzy VIKOR methods to calculate the importance of the indicators and rank the potential vendors. The findings showed that material costs, environmental performance evaluation, manufacture flexibility, service level, and system reliability were the most important criteria.

In [33], the application of agent technology on a combined problem of sustainable supplier selection and order allocation is presented. The developed and implemented multi-agent systems (MAS) approach in this paper demonstrates the contributions of agent technology in addressing the communication and information exchange challenges in vendor selection partnerships focusing specifically on the relationship between vendors and buyers. A real case study in the medical device sector supply chain is presented.

In all the above-mentioned papers [31,32,33] the brands of medical devices and the IoT connecting issues are not taken into account.

In [34], a policy and process for addressing the secure network connectivity of the medical devices in the inventory of Carilion Clinic was developed. Using a combination of industry best practices based on information technology infrastructure, Carilion’s technology services group has interfaced more than 700 patient monitors and over 200 point-of-care medical devices to their medical record. Medical device security and risk assessment are discussed as a part of the vendor selection and contracting process. Unfortunately, no model for vendor selection is described in this paper.

An analysis of recent papers, to our knowledge, shows that the problem of vendor selection in healthcare does not take into account the brand, the number of purchased items or communication features with some software platforms.

Our approach to the vendor selection problem is new. It uses a combination of a new type of BWM weighting method and an optimization model that provides a solution by taking into account the vendor, the brand and a combination of performance and cost. The proposed solution tries to approach the problem of purchasing SMD from another point of view and to combine specific approaches to the problem in a unified approach.

The problem of procurement of SMD in healthcare is of great complexity and the proposed solution takes into account multiple aspects, which are important in making a well-founded decision.

## 4. Best Worst Method and Flexible Best Worst Method (FBWM)

In the literature, there are several weighting methods. These methods are divided into subjective and objective methods. Subjective weighting methods are based on the expert’s opinion; whereas, the objective methods are based on the decision matrix. In our approach, we will consider a subjective weighting method. Examples of more often used subjective weighting methods are the analytical hierarchy process (AHP) [35,36], simple multi-attribute rating technique (SMART) [37], SMARTS [38], analytical network process (ANP) [39], step-wise weight assessment ratio analysis (SWARA) [40], best worst method (BWM) [41], weighted aggregated sum product assessment (WASPAS) [42] and extended step-wise weight assessment ratio analysis (SWARA) [43]. A summary of the criteria weighting methods is presented in [44].

Due to human limitations, due to the ability, experience and knowledge of an expert, it is difficult to compare several criteria or alternatives simultaneously. However, it is relatively easy to determine the dominance of one criterion over another. As such, a peer-to-peer comparison method is an important tool for making multi-criteria decisions.

From these weighting methods the best worst method (BWM) [41] was selected. BWM is one of the recent methods based on peer-to-peer comparison. It is based on the evaluations of the current criterion and the best criterion (the most important) and the worst criterion (the least important) [45]. In BWM, two criteria are chosen from a set of given criteria. One criterion is the best criterion, and the other criterion is the worst criterion. Then, pairwise comparisons are made for the best criterion with each criterion and a first vector of pairwise comparisons is created. The worst criterion is compared, in pairs, with each criterion and the second vector of comparisons in pairs is created. The comparisons are made on the basis of an evaluation scale with values from one to nine. In order to calculate the criteria weights, a mathematical programming model is solved. The optimal solutions are the criteria weights. The consistency of pairwise comparisons is verified. If the comparisons in pairs for the two vectors are not consistent, the evaluation is repeated, and the mathematical programming model is solved again. This process continues until a suitable consistency is reached.

Compared to the analytical hierarchy process (AHP), the analytic network process (ANP), and SMART (Simple Multi-Attribute Rating Technique), BWM has several advantages:

- By identifying the best and worst criteria before making pairwise comparisons between criteria, an expert already has a clearer understanding of criteria evaluation. This involves more consistent pairwise comparisons.
- BWM requires fewer pairwise comparisons than AHP, and thus the complexity and the time required for experts to evaluate the criteria is greatly reduced. If *n* is the number of criteria, then BWM requires two pairwise comparison vectors versus AHP which requires a *n* × *n* matrix of pairwise comparisons. For AHP, a number of *n*(*n* − 1)/2 pairwise comparisons are required; whereas, in BWM a number of 2*n* − 3 comparisons of the current criteria with the best and the worst criterion are required.
- BWM uses a simpler scale from one to nine; whereas, AHP uses a larger scale from 1/9; 1/8, …, 1, 2, …, 9. This gives an advantage to BWM over AHP because the number of comparisons is smaller.
- In AHP, the solution becomes inconsistent when CR—consistency ratio—is greater than 0.1. In this case, a need to revise the AHP matrix comparisons in order to improve (decrease) CR is necessary. Revising comparisons in the two vectors of BWM is a much easier task than revising comparisons from the matrix of pairwise comparisons in AHP.
- Only one comparison vector is used in the SMART method. This makes SMART very efficient in terms of the amount of data and time required for comparisons. The main weakness is that the consistency of the pairwise comparisons cannot be easily verified. The use of a pairwise comparison matrix in AHP offers the possibility to verify the consistency of the comparisons on pairs but is not efficient in terms of the number of comparisons and the time required for comparisons. A lot of information needs to be asked from the decision maker. BWM is an efficient method in terms of the number of comparisons and the time required for them. It also offers the possibility to check the consistency of comparisons in pairs.

BWM has been widely used to solve various problems in various fields. Some recent domains in which BWM has been applied include: blockchain technology in the automotive industry supply chain [46], renewable energy [47], industry 4.0 technologies [48], environmental performance evaluation [49], solar energy [50], management performance of small islands [51] and land evaluation [52]. Some recent group BWM applications were made in the following areas: robot selection [53], supplier selection [54,55], wind farm site selection [56], COVID-19 outbreak [57], cloud service providers selection [58] and green supplier selection [59].

A state-of-the-art survey on integrations and applications of the best worst method in decision making for the period from 2015 to January 2019 is presented in [60].

Based on the BWM method, we develop a flexible BWM method for our approach. We present the BWM method and our flexible BWM method in the following section.

### 4.1. The BWM Method

*Step 1*. A set *C* = {*C*_1_, *C*_2_,…, *C_n_*} of criteria is defined.

*Step 2*. The best criterion *C_B_* and the worst criterion *C_W_* are selected from set *C* of the SMD types criteria.

*Step 3*. The preference for the best criterion *C_B_* over the other criteria, using a scale of scores from 1 to 9, is determined by pairwise comparisons. A vector $a_{B}=\left(a_{Bj}\right);j=1,\;2,\ldots ,n$ is obtained. Here, $a_{Bj}$ denotes preference for criterion *C_B_* over criterion *C_j_*.

*Step 4*. The preference of all criteria over the worst criterion *C_W_* using a scale of scores from 1 to 9, is determined by pairwise comparisons. A vector $a_{W}=\left(a_{jW}\right);j=1,\;2,\ldots ,n$ is obtained. Here, $a_{jW}$ denotes preference for criterion *C_j_* over criterion *C_W_*.

*Step 5*. In order to obtain the most consistent weights with the pairwise comparisons, the following programming problem is considered:

(1) $$\{\begin{array}{c} \begin{array}{c} \min\left[\underset{1\leq j\leq n}{\max}\left(\left|a_{Bj}w_{j}-w_{B}\right|,\left|a_{jW}w_{W}-w_{j}\right|\right)\right]\\ \overset{n}{\underset{j=1}{\sum }}w_{j}=1 \end{array}\\ 0<w_{W}\leq w_{j}\leq w_{B}\;\mathrm{for}\;\mathrm{every}\;j=1,\;2,\ldots ,n \end{array}$$

*Step 6.* The above problem is again nonlinear because it contains absolute values. It can be transformed into an equivalent linear programming problem, as shown below.

(2) $$\{\begin{array}{c} \begin{array}{c} \begin{array}{c} \begin{array}{c} \begin{array}{c} \min\left[\xi \right]\\ -\xi \leq a_{Bj}w_{j}-w_{B}\leq \xi ,\;j=1,\;2,\ldots ,n \end{array}\\ -\xi \leq a_{jW}w_{W}-w_{j}\leq \xi ,\;j=1,\;2,\ldots ,n\; \end{array}\\ \overset{n}{\underset{j=1}{\sum }}w_{j}=1 \end{array}\\ 0<w_{W}\leq w_{j}\leq w_{B}\;\mathrm{for}\;\mathrm{every}\;j=1,2,\ldots ,n \end{array}\\ \xi \geq 0 \end{array}$$

In the above model, the decision variables are $w_{j}$, *j* = 1, 2, …, *n* and ξ.

The vector $w=\left(w_{1},w_{2},\ldots ,w_{n}\right)$ is the solution of the above linear programming problem.

### 4.2. Flexible Best Worst Method with Flexible Evaluation (FBWM)

In the classical BWM, the following constraint is made: $a_{Bj}$ and $a_{jW}$ belong to a finite set {1, 2, …, 9}. One can relax the above constraint supposing that $a_{Bj}$ and $a_{jW}$ are real numbers that belong to the interval [1,9].

In order to increase the consistency of the model, the following conditions should be satisfied:

1.
$a_{BB}=a_{WW}=1$

2. $1\leq a_{Bj}\leq 9$ and $1\leq a_{jW}\leq 9$ for every *j* = 1, 2, …, *n*
3.
$a_{BW}=\underset{j}{\max}a_{Bj}=\underset{j}{\max}a_{jW}$

4. $\left(a_{Bi}-a_{Bj}\;\right)\left(a_{iW}-a_{jW}\right)\leq 0$ for every i,j∈{1,2,…,n}

The formulation of the linear programming problem for the FBWM is the following:

(3) $$\{\begin{array}{c} \begin{array}{c} \min\left[\underset{1\leq j\leq n}{\max}\left(\left|a_{Bj}w_{j}-w_{B}\right|,\left|a_{jW}w_{W}-w_{j}\right|\right)\right]\\ \overset{n}{\underset{j=1}{\sum }}w_{j}=1 \end{array}\\ 0<w_{W}\leq w_{j}\leq w_{B}\;\mathrm{for}\;\mathrm{every}\;j\neq B,W \end{array}$$

One can easily see that the conclusions of the following theorem hold:

**Theorem 1.**  *Suppose that the system of evaluations in the FBWM is fully consistent, that is,* $a_{Bj}a_{jW}=a_{BW}$*for every j* = 1, 2, …, *n*. *Then, the following analytic formula for the weights holds*:

(4) $$w_{j}=\frac{1}{a_{Bj}\left(\sum_{r=1}^{n}\;\frac{1}{a_{Br}}\right)}\;\mathrm{for}\;\mathrm{every}\;j=1,\;2,\;\ldots ,\;n$$

From the above theorem one can easily see that if the evaluation vector $a_{B}=\left(a_{B1},a_{B2},\ldots ,a_{Bn}\right)$ is given, then we can compute the criteria weights for a fully consistent set of evaluations with the Formula (4). In this case, the entries of the vector $a_{W}=\left(a_{1W},a_{2W},\ldots ,a_{nW}\right)$ do need not to be integers.

## 5. A Hybrid Multi-Criteria Approach for Vendor Selection

The healthcare manager has to purchase a set of SMD types with capacity to communicate with an IoT platform. The manager wants to buy a number of SMD types that meet their preferences from a set of vendors. In our paper, the vendor is a retailer. The vendor’s offer has SMD belonging to various brands. An important problem is the SMD type, brand and vendor’s evaluation and performance calculation. This is a multi-criteria decision problem because several criteria are involved in the selection of SMD type, brand and vendor’s portfolio.

When making a choice, the manager has two objectives: to maximize the performance and to minimize the cost of SMD that they purchase. This is a multi-objective optimization problem. The manager has to take into account that these medical devices can be connected to an IoT platform.

In order to solve the vendor selection problem, we shall develop a hybrid multi-criteria approach that combines a performance evaluation model and a multi-objective model.

The decision approach of our hybrid method has four stages:

- The definition of the decision problem and the input data;
- Computation of criteria weights for SMD, brands and vendors. Calculus is based on a flexible best worst method;
- The performance evaluation for SMD, brands and vendors;
- Solving a multi-objective optimization model and finding the optimal SMD, brands and vendors.

*Stage 1. The definition of the decision problem and input data*

In the first stage, the definition of the decision problem is made. A set of SMD, a set of vendors and a set of brands are introduced.

The input data for the decision approach, all stages, are:

- The set *D* = {$D_{1},D_{2},\ldots ,D_{n}$} of SMD types;
- The set *B* = {$B_{1},B_{2},\ldots ,B_{m}$} of brands;
- The set *V* = {$V_{1},V_{2},\ldots ,V_{p}$} of vendors.

This data is used in all the following stages.

*Stage 2. Computation of criteria weights for SMD, brands and vendors*

In the second stage, criteria weights for SMD, brands and vendors are determined starting from evaluations and using a flexible version of the best worst (FBWM) method.

The input data for the second stage are:

- The set $C=\{C_{1},C_{2},\ldots ,C_{o}$} of SMD types criteria;
- The set $\bar{C}=\{{\bar{C}}_{1},{\bar{C}}_{2},\ldots ,{\bar{C}}_{r}$} of brands criteria;
- The set $\overset{=}{C}=\{{\overset{=}{C}}_{1},{\overset{=}{C}}_{2},\ldots ,{\overset{=}{C}}_{s}$} of vendor’s criteria;
- Scale of scores from 1 to 9 (denotes preference).

We applied the FBWM for the sets of criteria C, $\bar{C}$, and $\overset{=}{C}$.

*Step 1.* The best criterion $C_{B}$ and the worst criterion $C_{w}$ are selected from the set C of SMD types criteria.

The best criterion ${\bar{C}}_{B}$ and the worst criterion ${\bar{C}}_{w}$ are selected from the set $\bar{C}$ of brands criteria.

The best criterion ${\overset{=}{C}}_{B}$ and the worst criterion ${\overset{=}{C}}_{w}$ are selected from the set $\overset{=}{C}$ of vendor’s criteria.

*Step 2.* The SMD preference of the best criterion $C_{B}$ over the other criteria, using a scale of scores from 1 to 9, is determined by pairwise comparisons. A vector $a_{B}=\left(a_{Bl}\right);l=1,2,\ldots ,o$ is obtained. Here, $a_{Bl}$ denotes preference of criterion $C_{B}$ over criterion $C_{l}$.

The brands preference of the best criterion ${\bar{C}}_{B}$ over the other criteria, using the same scale from 1 to 9, is determined by pairwise comparisons. A vector ${\bar{a}}_{B}=\left({\bar{a}}_{Bu}\right);u=1,2,\ldots ,r$ is obtained. Here, ${\bar{a}}_{Bu}$ denotes preference of criterion ${\bar{C}}_{B}$ over criterion ${\bar{C}}_{u}$.

The vendors’ preference of the best criterion ${\overset{=}{C}}_{B}$ over the other criteria, using the same scale from 1 to 9, is determined by pairwise comparisons. A vector ${\overset{=}{a}}_{B}=\left({\overset{=}{a}}_{Bv}\right);v=1,2,\ldots ,s$ is obtained. Here, ${\overset{=}{a}}_{Bv}$ denotes preference of criterion ${\overset{=}{C}}_{B}$ over criterion ${\overset{=}{C}}_{v}$.

*Step 3.* FBWM supposes that the SMD evaluations, brand evaluations and vendor’s evaluations are fully consistent, that is:

- $a_{lW}=\frac{a_{BW}}{a_{Bl}}$; *l* = 1, 2, …, *o*, for every SMD, where $a_{BW}=\underset{l}{\max}a_{Bl}$;
- ${\bar{a}}_{uW}=\frac{{\bar{a}}_{BW}}{{\bar{a}}_{Bu}}$; *u* = 1, 2, …, *r*, for every brand, where ${\bar{a}}_{BW}=\underset{u}{\max}{\bar{a}}_{Bu}$;
- ${\overset{=}{a}}_{vW}=\frac{{\bar{a}}_{BW}}{{\bar{a}}_{Bv}}$; *v* = 1, 2, …, *s*, for every vendor, where ${\overset{=}{a}}_{BW}=\underset{v}{\max}{\overset{=}{a}}_{Bv}$.

Then, the SMD criteria weights $w=\left(w_{l}\right);l=1,2,\ldots ,o\;$, brand criteria weights $\bar{w}=\left({\bar{w}}_{u}\right);u=1,2,\ldots ,r$ and vendor’s criteria weights $\overset{=}{w}=\left({\overset{=}{w}}_{v}\right);v=1,2,\ldots ,s$ are obtained:

(5) $$w_{l}=\frac{1}{a_{Bl}\left(\sum_{y=1}^{o}\;\frac{1}{a_{By}}\right)}\;,\;l=1,2,\ldots ,o$$

(6) $${\bar{w}}_{u}=\frac{1}{{\bar{a}}_{Bu}\left(\sum_{y=1}^{r}\;\frac{1}{{\bar{a}}_{By}}\right)}\;,\;u=1,2,\ldots ,r$$

(7) $${\overset{=}{w}}_{v}=\frac{1}{{\overset{=}{a}}_{Bv}\left(\sum_{y=1}^{s}\;\frac{1}{{\overset{=}{a}}_{By}}\right)},\;v=1,2,\ldots ,s$$

*Stage 3. The performance of SMD, brands and vendors*

In this stage, the performances of SMD types, vendors and brands are calculated.

The input data for the third stage are:

- the criteria weights vectors $w,\;\bar{w},\;\overset{=}{w}$ that were calculated in the second stage;
- the evaluation scale composed from integer numbers between 1 and 10 (1 for the worst and 10 for the best).

*Step 1.* The SMD evaluation matrix $T=\left(t_{il}\right),\;i=1,2,\ldots ,n;l=1,2,\ldots ,o$ is built. The entry $t_{il}$ is the evaluation of the *i*-th SMD for criterion *C_l_*.

*Step 2.* The brand evaluation matrix $\bar{T}=\left({\bar{t}}_{ju}\right),\;j=1,2,\ldots ,m;u=1,2,\ldots ,r$ is built. The entry ${\bar{t}}_{ju}$ is the evaluation of brand *B_j_* for criterion ${\bar{C}}_{u}$.

*Step 3.* The vendor’s evaluation matrix $\overset{=}{T}=\left({\overset{=}{t}}_{kv}\right),\;k=1,2,\ldots ,p;v=1,2,\ldots ,s$ is built. The entry ${\overset{=}{t}}_{kv}$ is the evaluation of vendor $V_{k}$ for criterion ${\overset{=}{C}}_{v}$.

*Step 4.* The matrices $T,\;\bar{T}$ and $\overset{=}{T}$ are normalized using the max normalization method. The normalized matrices are $T^{N}=\left(t_{il}^{N}\right),\;{\bar{T}}^{N}=\left({\bar{t}}_{ju}^{N}\right)\;\mathrm{and}\;{\overset{=}{T}}^{N}=\left({\overset{=}{t}}_{kv}^{N}\right)$ where:

(8) $$t_{il}^{N}=\frac{t_{il}}{t_{l}^{max}};\;{\bar{t}}_{ju}^{N}=\frac{{\bar{t}}_{ju}}{{\bar{t}}_{u}^{max}};\;{\overset{=}{t}}_{kv}^{N}=\frac{{\overset{=}{t}}_{kv}}{{\overset{=}{t}}_{v}^{max}}$$

where $t_{l}^{max}=\underset{i}{\max}t_{il}$; ${\bar{t}}_{u}^{max}=\underset{j}{\max}{\bar{t}}_{ju}$ and ${\overset{=}{t}}_{v}^{max}=\underset{k}{\max}{\overset{=}{t}}_{kv}$.

*Step 5.* The SMD performance $q^{D}=\left(q_{i}^{D}\right)$, brand performance $q^{B}=(q_{k}^{B})\;\mathrm{and}$ vendors’ performance $q^{V}=(q_{j}^{V})$ are obtained:

(9) $$q_{i}^{D}=\overset{o}{\underset{l=1}{\sum }}w_{l}*t_{il}^{N};\;q_{j}^{B}=\overset{s}{\underset{u=1}{\sum }}{\bar{w}}_{u}*{\bar{t}}_{ju}^{N};\;q_{k}^{V}=\overset{r}{\underset{v=1}{\sum }}{\overset{=}{w}}_{v}*{\overset{=}{t}}_{kv}^{N}$$

*Step 6.* The cumulative performance for SMD, vendors and brands $Q=(q_{ijk})$ is calculated:

(10) $$q_{ijk}=q_{i}^{D}*q_{j}^{B}*q_{k}^{V}$$

Steps 4 and 5 correspond to the multi-attribute method SAW—simple additive weighting.

*Stage 4. Vendor selection based on a multi-objective model*

In the fourth stage, the SMD performances found in the third stage are used to formulate a multi-objective optimization model for the vendor selection. The model has two objective functions: cost minimization and performance maximization. Starting from the multi-objective model for vendor selection, we formulate a single objective model, which includes a budget for the purchasing process.

The input data are:

- *S*—The sum of money to be invested;
- $d^{min}=\left(d_{i}^{min}\right)$ where $d_{i}^{min}$ is the lower bound for number of devices of type *D_i_* that have to be bought;
- $d^{max}=\left(d_{i}^{max}\right)$ where $d_{i}^{max}$ is the upper bound for the number of devices of type *D_i_* that have to be bought;
- **D** = (*d_ijk_*) where *d_ijk_* is the upper bound for the number of devices of type *D_i_* belonging to brand *B_j_* available for selling at vendor *V_k_*;
- $C=\left(c_{ijk}\right)$ where $c_{ijk}$ is the cost of a device of type *D_i_* belonging to brand *B_j_* at vendor *V_k_*;
- **Q** = (*q_ijk_*) where *q_ijk_* is the performance of device of type *D_i_* belonging to brand *B_j_* sold by vendor *V_k_* (obtained in stage three).

*Step 1.* The matrix C is normalized using the max normalization method. The normalized matrix is $C^{N}=\left(c_{ijk}^{N}\right)$ where $c_{ijk}^{N}=\frac{c_{ijk}}{c_{k}^{max}}$ where $c_{k}^{max}=\underset{ij}{\max}c_{ijk}$.

*Step 2.* The following condition is necessary for the existence of feasible solutions:

(11) $$\sum_{j=1}^{m}\sum_{k=1}^{p}d_{ijk}\geq d_{i}^{min},\;i=1,2,\ldots ,n$$

The validity of condition (11) should be verified.

*Step 3.* An important problem in solving the optimization model for buying SMD is the determination of the range of parameter *S*. The lower and upper bounds for the parameter *S* can be determined by solving the following two optimization problems:

(12) $$\{\begin{array}{c} \min\left[\overset{n}{\underset{i=1}{\sum }}\overset{m}{\underset{j=1}{\sum }}\overset{p}{\underset{k=1}{\sum }}c_{ijk}*x_{ijk}\right]\\ d_{i}^{min}\leq \overset{m}{\underset{j=1}{\sum }}\overset{p}{\underset{k=1}{\sum }}x_{ijk}\leq d_{i}^{max},\;i=1,2,\ldots ,n\\ x_{ijk}\leq d_{ijk},\;i=1,2,\ldots ,n,\;j=1,2,\ldots ,m,\;k=1,2,\ldots ,p\\ x_{ijk}\in N,\;i=1,2,\ldots ,n,\;j=1,2,\ldots ,m,\;k=1,2,\ldots ,p \end{array}$$

and

(13) $$\{\begin{array}{c} \max\left[\overset{n}{\underset{i=1}{\sum }}\overset{m}{\underset{j=1}{\sum }}\overset{p}{\underset{k=1}{\sum }}c_{ijk}*x_{ijk}\right]\\ d_{i}^{min}\leq \overset{m}{\underset{j=1}{\sum }}\overset{p}{\underset{k=1}{\sum }}x_{ijk}\leq d_{i}^{max},\;i=1,2,\ldots ,n\\ x_{ijk}\leq d_{ijk},\;i=1,2,\ldots ,n,\;j=1,2,\ldots ,m,\;k=1,2,\ldots ,p\\ x_{ijk}\in N,\;i=1,2,\ldots ,n,\;j=1,2,\ldots ,m,\;k=1,2,\ldots ,p \end{array}$$

Denote by *S*_1_ (respectively by *S*_2_) the optimal value of the problem (12) (respectively of the problem (13)). *S* should be chosen in the interval [*S*_1_, *S*_2_].

*Step 4.* The formulation of the multi-objective optimization model for buying SMD is:

(14) $$\{\begin{array}{c} \min\left[\overset{n}{\underset{i=1}{\sum }}\overset{m}{\underset{j=1}{\sum }}\overset{p}{\underset{k=1}{\sum }}c_{ijk}^{N}*x_{ijk}\right]\\ \max\left[\overset{m}{\underset{j=1}{\sum }}\overset{p}{\underset{k=1}{\sum }}q_{ijk}*x_{ijk}\right],\;i=1,2,\ldots ,n\\ d_{i}^{min}\leq \overset{m}{\underset{j=1}{\sum }}\overset{p}{\underset{k=1}{\sum }}x_{ijk}\leq d_{i}^{max},\;i=1,2,\ldots ,n\\ x_{ijk}\leq d_{ijk},\;i=1,2,\ldots ,n,\;j=1,2,\ldots ,m,\;k=1,2,\ldots ,p\\ x_{ijk}\in N,\;i=1,2,\ldots ,n,\;j=1,2,\ldots ,m,\;k=1,2,\ldots ,p \end{array}$$

The decision variable of the multi-objective optimization model for buying SMD is the matrix $X=\left(x_{ijk}\right)$, *i =* 1, 2, *…*, *n*; *j =* 1, 2, …, *m*; *k =* 1, 2, …, *p*. Here, $x_{ijk}$ represents the number of SMD of type *D_i_* belonging to brand *B_j_* bought from vendor *V_k_*.

Starting from the multi-objective optimization model (14), we formulate a single objective model, the trade-off cost-performance model. The optimization model is:

(15) $$\{\begin{array}{c} \min\left[\overset{n}{\underset{i=1}{\sum }}\overset{m}{\underset{j=1}{\sum }}\overset{p}{\underset{k=1}{\sum }}[\left(1-\lambda \right)c_{ijk}^{N}-\lambda q_{ijk}\right]x_{ijk}\\ \overset{n}{\underset{i=1}{\sum }}\overset{m}{\underset{j=1}{\sum }}\overset{p}{\underset{k=1}{\sum }}c_{ijk}*x_{ijk}\leq S\\ d_{i}^{min}\leq \overset{m}{\underset{j=1}{\sum }}\overset{p}{\underset{r=1}{\sum }}x_{ijr}\leq d_{i}^{max},\;i=1,2,\ldots ,n\\ x_{ijk}\leq d_{ijk},\;i=1,2,\ldots ,n,\;j=1,2,\ldots ,m,\;k=1,2,\ldots ,p\\ x_{ijk}\in N,\;i=1,2,\ldots ,n,\;j=1,2,\ldots ,m,\;k=1,2,\ldots ,p \end{array}$$

In the optimization model (15), the value of parameter *λ* can be chosen in the interval [0, 1]. The greater the value of *λ,* the greater the importance of the SMD performance for the user versus the SMD cost. For λ = 0, only the SMD cost minimization model will be considered and for λ = 1, only the SMD performance maximization model will be considered.

Solve the single optimization model (15) and find the optimal solution.

## 6. Case Study

In the following section, we shall apply the above-described hybrid multi-criteria model to the procurement of a set of SMD for a healthcare center.

*Stage 1*

The manager wants to buy five SMD types: activity tracker (*D*_1_), blood pressure monitor (*D*_2_), pulse oximeter (*D*_3_), body weight scale (*D*_4_) and glucometer (*D*_5_). For these SMD, three brands *B*_1_, *B*_2_ and *B*_3_ and five vendors *V*_1_, *V*_2_, *V*_3_, *V*_4_ and *V*_5_ are selected.

The SMD set is *D* = {*D*_1_, *D*_2_, *D*_3_, *D*_4_, *D*_5_}, the brands set is *B* = {*B*_1_, *B*_2_, *B*_3_} and the vendors set is *V* = {*V*_1_, *V*_2_, *V*_3_, *V*_4_, *V*_5_}.

On the market, an offer from a variety of vendors that sell SMD from different brands with experience in the field is available. Prominent brands in the global IoT medical devices market in a top 10 by revenue [61] are: Siemens AG, Abbott Laboratories, Honeywell Life Care Solutions, Medtronic Plc, Boston Scientific Corporation, GE Healthcare, Omron Corporation, Biotronik, Johnson & Johnson and Philips Healthcare.

*Stage 2*

In the second stage of the hybrid multi-criteria model, criteria weights for the SMD, brands and vendors are calculated using FBWM.

For comparison, the BWM and FBWM methods are applied to calculate the weights of the SMD criteria. The best criterion and the worst criterion are chosen. Then, the best and worst criteria are compared with all the other criteria. The SMD criteria and the corresponding symbols are presented in Table 1.

The best criterion is selected as SMD type quality ($C_{1}$) and the worst criterion is selected as SMD type comfort ($C_{3}$). In order to make the comparison between the BWM and FBWM methods, integer numbers (on a scale from one to nine) are used.

**Figure 3:**
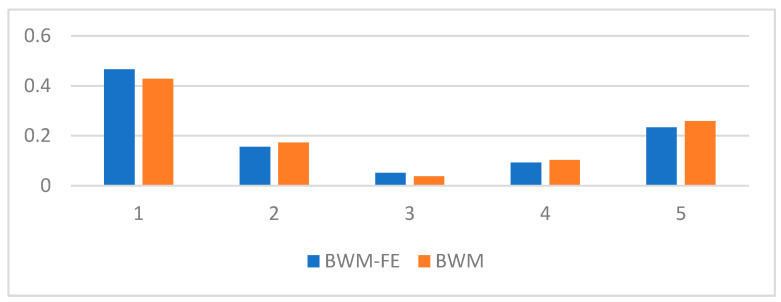
SMD criteria weights comparison.

The comparison between the SMD criteria weights computed using the BWM and FBWM methods are presented in Table 2 and Figure 3. The BWM uses the SMD criteria weights and the vectors from the third and the fourth column of Table 2 for computing. The FBWM uses the SMD criteria weights and only the vector from the third column of Table 2 for computing.

The brands criteria and the corresponding symbols are presented in Table 3.

**Figure 4:**
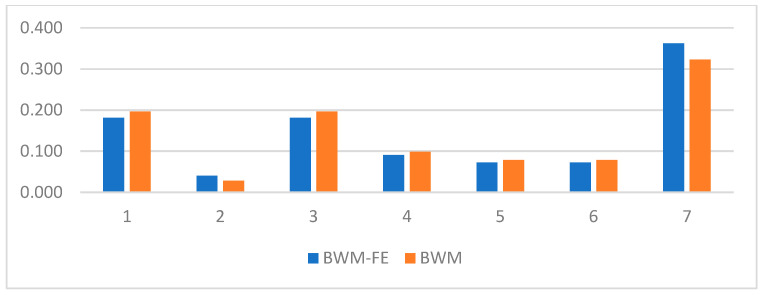
Brand criteria weights comparison.

The best criterion is selected as brand experience in SMD type (${\bar{C}}_{7}$) and the worst criterion is selected as brand financial status (${\bar{C}}_{2}$). The comparison between the brands criteria weights computed using the BWM and FBWM methods is presented in Table 4 and Figure 4.

The vendors’ criteria and the corresponding symbols are presented in Table 5.

**Figure 5:**
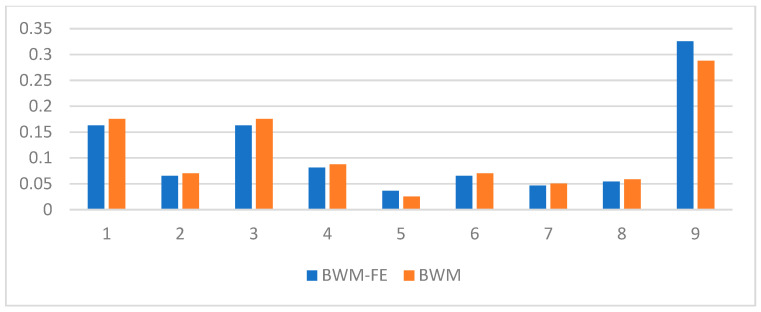
Vendors’ criteria weights comparison.

The best criterion is selected as reputation (${\overset{=}{C}}_{9}$) and the worst criterion is selected as capacity (${\overset{=}{C}}_{5}$). The comparison between the vendors’ criteria weights computed using the BWM and FBWM methods is presented in Table 6 and Figure 5.

One can easily see that the weights obtained for all three cases using the FBWM method are similar to those obtained using the BWM method. Recall that FBWM used fewer pairwise comparisons than BWM.

*Stage 3*

The SMD evaluation matrix $T=\left(t_{il}\right),\;i=1,\;2,\ldots ,\;5;l=1,\;2,\ldots ,\;5$, the brands evaluation matrix $\bar{T}=\left({\bar{t}}_{ju}\right),\;j=1,\;2,\ldots ,3;u=1,\;2,\ldots ,\;7$ and the vendor’s evaluation matrix $\overset{=}{T}=\left({\overset{=}{t}}_{kv}\right),\;k=1,\;2,\ldots ,\;5;v=1,\;2,\ldots ,\;9$ are built. In Table 7 the matrix **T** is presented.

The matrices $T,\;\bar{T}$ and $\overset{=}{T}$ are normalized and weighted.

The performances of SMD types, vendors and brands are calculated based on Equation (9) (Table 8).

The cumulative performance for SMD, vendors and brands $Q=(q_{ijk}),\;i=1,\;2,\ldots ,\;5;j=1,\;2,\ldots ,3;k=1,\;2,\ldots ,5$ is calculated based on Equation (10). See the performance values in Table 9.

*Stage 4*

The vectors whose arrays are the lower bound and the upper bound for the SMD number of pieces $d^{min}$ and $d^{max}$ that have to be bought are presented in Table 10.

The matrix **D**—whose arrays are the upper bound for the number of SMD types from set *D*—belonging to a brand from set *B* available for selling at a vendor from set *V* is presented in Table 11.

The matrix C —whose arrays are the costs of SMD types from set *D*—belonging to a brand from set *B* available for selling at a vendor from set *V* is presented in Table 12.

The matrix C is normalized and the validity of condition (11) is verified (Table 13). The total number of SMD types, brands and vendors is greater than the entries of the vector $d^{min}$**.**

In Table 13, the validity of condition (11), that is, for each type of SMD the total number of SMD is greater than the lower bound **d^*min*^**, is checked.

The lower and upper bounds for the parameter *S* are determined by solving optimization problems (12) and (13). The optimal value of problem (12) is *S*_1_ = EUR 60759. The optimal solution of problem (13) is *S*_2_ = EUR 83445.

*S* is chosen as follows *S* = *S*_2_ = EUR 83445.

The optimal solutions obtained by solving optimization problem (15) and the variation of the *λ* parameter in the interval [0, 1] with step 0.1 are presented in Table 14.

From Table 14, one can see that the solution with the minimum cost (λ = 0) is the following (see column 1): 25 SMD of type 1, purchased from brand *B*_1_, vendor *V*_3_, 50 SMD of type 1, purchased from brand *B*_2_, vendor *V*_3_, …, 35 SMD of type 5, purchased from brand *B*_1_, vendor *V*_3_ and 90 SMD of type 5 purchased from brand *B*_3_, vendor *V*_3_. The important vendors are *V*_1_ and *V*_3_.

In the case that the manager is only interested in SMD performance, we are in the case λ = 1. The number of SMD types, brands and vendors can be found in the last column of Table 14. The important vendors are *V*_2_, *V*_3_ and *V*_4_.

In the case that cost and performance have the same importance, then the number of SMD types, brands and vendors can be found in the column of Table 14 corresponding to the value of λ equal to 0.5.

The solutions of the optimization problems were obtained using GAMS software, MIP—GNU Linear Programming Kit (GLPK) solver. GLPK uses the revised simplex method and the primal-dual interior point method for non-integer problems and the branch-and-bound algorithm together with Gomory’s mixed integer cuts for (mixed) integer problems.

For λ = 0 only the cost minimization model will be solved and for λ = 1 only the performance maximization model will be solved.

The proposed total number of SMD to be purchased for each type and each value of the parameter λ, in the range [0, 1], is displayed in Table 15.

For example, for λ = 0.7 200 SMD of type 1, 125 SMD of type 2, 130 SMD of type 3, 145 SMD of type 4 and 135 SMD of type 5 are proposed to be purchased.

From Table 15, one can easily see that when the cost is the unique selection criterion (the case λ = 0), then the proposed number of SMD to be purchased is the minimum. As the performance is taken into account then the proposed number of SMD to be purchased increases.

## 7. Conclusions

The subject of our paper was motivated by the complexity of the SMD procurement decision process in healthcare. This domain deals with a great diversity of medical devices to be purchased, from different brands and vendors, which have an impact on the costs involved and health risks for patients in many ways. Through health applications, the delivery of sensors and healthcare medical devices can be improved. These technologies can help with lowering costs, facilitating the delivery of healthcare, and connecting people to medical services. In this paper, a survey of medical devices and medical sensors is presented in the context of IoT. There are stationary medical devices, wearable external medical devices, implantable medical devices and other medical devices. A decision support approach for SMD procurement is proposed to help decision makers from healthcare centers. Because of the complex structure of the decision process, a hybrid approach is proposed. The criteria weights are calculated with the help of a flexible version of the best worst method (FBWM) that reduces the number of evaluations made by the decision maker. A multi-objective model is defined for solving the vendor selection problem that takes into account the types of SMD, the brands and the vendors’ offers. The proposed hybrid approach is illustrated by a case study for the procurement of a set of SMD for a healthcare center. A comparison between BWM and FBWM is made for three cases of criteria weights calculation. The results obtained using FBWM are similar to those obtained using the BWM method taking into account that FBWM used fewer pairwise comparisons than BWM.

## Figures and Tables

**Table 1:** The SMD criteria and symbols.

Nr.crt.	SMD Criteria	Criteria
1	SMD type quality	$C_{1}$
2	Communication capacity with a IoT platform	$C_{2}$
3	SMD type comfort	$C_{3}$
4	SMD type safety	$C_{4}$
5	SMD type price	$C_{5}$

**Table 2:** Comparison between the SMD type criteria weights obtained with BWM and FBWM.

Nr.crt.	Criteria	$C_{1}$ Preference over All Criteria	Preference for All Criteria over the $C_{3}$	BWM Criteria Weights	FBWM Criteria Weights
1	$C_{1}$	1	9	0.428	0.466
2	$C_{2}$	3	7	0.173	0.155
3	$C_{3}$	9	1	0.038	0.052
4	$C_{4}$	5	5	0.104	0.093
5	$C_{5}$	2	8	0.259	0.233

**Table 3:** The brands criteria and symbols.

Nr.crt.	Brands Criteria	Criteria
1	Brand reputation	${\bar{C}}_{1}$
2	Brand financial status	${\bar{C}}_{2}$
3	Experience with brand	${\bar{C}}_{3}$
4	Market analysis	${\bar{C}}_{4}$
5	Technical ability and flexibility	${\bar{C}}_{5}$
6	Quality management system certification	${\bar{C}}_{6}$
7	Brand experience in SMD type	${\bar{C}}_{7}$

**Table 4:** Comparison between the brand criteria weights obtained using BWM and FBWM.

Nr.crt.	Criteria	${\bar{C}}_{7}$ Preference over All Criteria	Preference for All Criteria over the ${\bar{C}}_{2}$	BWM Criteria Weights	FBWM Criteria Weights
1	${\bar{C}}_{1}$	2	8	0.181	0.197
2	${\bar{C}}_{2}$	9	1	0.040	0.028
3	${\bar{C}}_{3}$	2	8	0.181	0.197
4	${\bar{C}}_{4}$	4	6	0.091	0.098
5	${\bar{C}}_{5}$	5	5	0.072	0.079
6	${\bar{C}}_{6}$	5	5	0.072	0.079
7	${\bar{C}}_{7}$	1	9	0.362	0.323

**Table 5:** The vendor’s criteria and symbols.

Nr.crt.	Vendors Criteria	Criteria
1	Delivery cost	${\overset{=}{C}}_{1}$
2	Payment terms	${\overset{=}{C}}_{2}$
3	Warranty	${\overset{=}{C}}_{3}$
4	On-time delivery	${\overset{=}{C}}_{4}$
5	Capacity	${\overset{=}{C}}_{5}$
6	Training after purchasing	${\overset{=}{C}}_{6}$
7	Transport quality	${\overset{=}{C}}_{7}$
8	Digitalisation	${\overset{=}{C}}_{8}$
9	Reputation	${\overset{=}{C}}_{9}$

**Table 6:** Comparison between the vendors’ criteria weights obtained using BWM and FBWM.

Nr.crt.	Criteria	${\overset{=}{C}}_{9}$ Preference over All Criteria	Preference for All Criteria over the ${\overset{=}{C}}_{5}$	BWM Criteria Weights	FBWM Criteria Weights
1	${\overset{=}{C}}_{1}$	2	8	0.163	0.175
2	${\overset{=}{C}}_{2}$	5	5	0.065	0.070
3	${\overset{=}{C}}_{3}$	2	8	0.163	0.175
4	${\overset{=}{C}}_{4}$	4	6	0.081	0.088
5	${\overset{=}{C}}_{5}$	9	1	0.036	0.025
6	${\overset{=}{C}}_{6}$	5	5	0.065	0.070
7	${\overset{=}{C}}_{7}$	7	3	0.047	0.050
8	${\overset{=}{C}}_{8}$	6	4	0.054	0.058
9	${\overset{=}{C}}_{9}$	1	9	0.326	0.288

**Table 7:** The evaluation matrix **T**.

SMD	$C_{1}$	$C_{2}$	$C_{3}$	$C_{4}$	$C_{5}$
*D*_1_	8	9	8	7	9
*D*_2_	9	9	7	8	8
*D*_3_	8	8	8	6	8
*D*_4_	7	8	7	8	7
*D*_5_	10	9	7	7	7

**Table 8:** The performances of SMD types, vendors and brands.

SMD	Brands	Vendors
0.895	0.836	0.839
0.921	1.000	0.905
0.840	0.909	0.940
0.785		0.907
0.930		0.846

**Table 9:** The cumulative performance.

SMD·Brand	Vendor				
	*V*_1_	*V*_2_	*V*_3_	*V*_4_	*V*_5_
*D*_1_·*B*_1_	0.628	0.677	0.703	0.679	0.633
*D*_1_·*B*_2_	0.751	0.810	0.841	0.812	0.757
*D*_1_·*B*_3_	0.683	0.736	0.764	0.738	0.688
*D*_2_·*B*_1_	0.646	0.697	0.723	0.698	0.651
*D*_2_·*B*_2_	0.773	0.834	0.865	0.835	0.779
*D*_2_·*B*_3_	0.703	0.758	0.786	0.759	0.708
*D*_3_·*B*_1_	0.589	0.636	0.660	0.637	0.594
*D*_3_·*B*_2_	0.705	0.761	0.790	0.762	0.710
*D*_3_·*B*_3_	0.641	0.691	0.717	0.693	0.646
*D*_4_·*B*_1_	0.550	0.594	0.616	0.595	0.554
*D*_4_·*B*_2_	0.659	0.710	0.737	0.712	0.663
*D*_4_·*B*_3_	0.598	0.645	0.670	0.647	0.603
*D*_5_·*B*_1_	0.653	0.704	0.730	0.705	0.657
*D*_5_·*B*_2_	0.781	0.842	0.874	0.844	0.786
*D*_5_·*B*_3_	0.709	0.765	0.794	0.767	0.715

**Table 10:** The minimum and maximum SMD number of pieces.

	*D*_1_	*D*_2_	*D*_3_	*D*_4_	*D*_5_
$d^{min}$	183	97	100	132	125
$d^{max}$	200	125	130	145	135

**Table 11:** The upper bound for the number of SMD types.

SMD·Brand	Vendor				
	*V*_1_	*V*_2_	*V*_3_	*V*_4_	*V*_5_
*D*_1_·*B*_1_	45	30	25	35	25
*D*_1_·*B*_2_	55	90	50	20	15
*D*_1_·*B*_3_	100	105	90	100	117
*D*_2_·*B*_1_	45	87	65	90	47
*D*_2_·*B*_2_	97	90	105	80	105
*D*_2_·*B*_3_	120	100	115	100	110
*D*_3_·*B*_1_	70	70	75	65	80
*D*_3_·*B*_2_	95	60	97	40	0
*D*_3_·*B*_3_	145	100	200	100	150
*D*_4_·*B*_1_	60	40	65	47	40
*D*_4_·*B*_2_	50	0	100	80	80
*D*_4_·*B*_3_	100	70	95	70	125
*D*_5_·*B*_1_	54	55	60	67	65
*D*_5_·*B*_2_	67	75	87	85	77
*D*_5_·*B*_3_	123	100	90	130	110

**Table 12:** The cost of SMD type (in EUR).

SMD·Brand	Vendor				
	*V*_1_	*V*_2_	*V*_3_	*V*_4_	*V*_5_
*D*_1_·*B*_1_	25	23	20	24	27
*D*_1_·*B*_2_	27	25	22	24	26
*D*_1_·*B*_3_	23	25	23	25	25
*D*_2_·*B*_1_	100	95	97	98	95
*D*_2_·*B*_2_	90	90	110	95	97
*D*_2_·*B*_3_	110	100	97	94	90
*D*_3_·*B*_1_	94	95	100	99	95
*D*_3_·*B*_2_	89	89	99	95	97
*D*_3_·*B*_3_	89	87	95	97	97
*D*_4_·*B*_1_	200	220	215	217	210
*D*_4_·*B*_2_	210	210	217	215	220
*D*_4_·*B*_3_	250	240	255	210	219
*D*_5_·*B*_1_	110	99	100	97	99
*D*_5_·*B*_2_	100	100	105	99	100
*D*_5_·*B*_3_	105	98	97	100	110

**Table 13:** The validity of condition (11).

SMD	*D*_1_	*D*_2_	*D*_3_	*D*_4_	*D*_5_
$d^{min}$	183	97	100	132	125
Total number of SMD belonging to brands and vendors	902	1356	1347	1022	1245

**Table 14:** The optimal solution for various values of *λ*.

SMD·Brand· Vendor	*λ*										
	0	0.1	0.2	0.3	0.4	0.5	0.6	0.7	0.8	0.9	1
*D*_1_·*B*_1_·*V*_3_	25	25	0	0	0	0	0	0	0	0	0
*D*_1_·*B*_2_·*V*_2_	0	18	60	90	90	90	90	90	90	90	90
*D*_1_·*B*_2_·*V*_3_	50	50	50	50	50	50	50	50	50	50	50
*D*_1_·*B*_2_·*V*_4_	0	0	0	0	20	20	20	20	20	20	20
*D*_1_·*B*_3_·*V*_1_	18	0	0	0	0	0	0	0	0	0	0
*D*_1_·*B*_3_·*V*_3_	90	90	90	60	40	40	40	40	40	40	40
*D*_2_·*B*_2_·*V*_1_	97	97	7	7	35	0	0	0	0	0	0
*D*_2_·*B*_2_·*V*_2_	0	0	90	90	90	90	90	25	25	25	0
*D*_2_·*B*_2_·*V*_3_	0	0	0	0	0	35	35	100	100	100	100
*D*_3_·*B*_2_·*V*_1_	0	95	95	0	0	0	0	0	0	0	0
*D*_3_·*B*_2_·*V*_2_	0	0	5	60	33	33	33	33	33	33	0
*D*_3_·*B*_2_·*V*_3_	0	0	0	40	97	97	97	97	97	97	97
*D*_3_·*B*_2_·*V*_4_	0	0	0	0	0	0	0	0	0	0	33
*D*_3_·*B*_3_·*V*_1_	100	5	0	0	0	0	0	0	0	0	0
*D*_4_·*B*_1_·*V*_1_	60	60	60	0	0	0	0	0	0	0	0
*D*_4_·*B*_1_·*V*_3_	22	0	0	0	0	0	0	0	0	0	0
*D*_4_·*B*_2_·*V*_1_	50	50	0	32	32	32	45	45	0	0	0
*D*_4_·*B*_2_·*V*_3_	0	22	72	100	100	100	100	100	100	100	100
*D*_4_·*B*_2_·*V*_4_	0	0	0	0	0	0	0	0	45	45	45
*D*_5_·*B*_1_·*V*_3_	35	35	0	0	0	0	0	0	0	0	0
*D*_5_·*B*_2_·*V*_2_	0	0	0	0	0	48	48	48	48	48	0
*D*_5_·*B*_2_·*V*_3_	0	0	35	87	87	87	87	87	87	87	87
*D*_5_·*B*_2_·*V*_4_	0	0	0	0	0	0	0	0	0	0	48
*D*_5_·*B*_3_·*V*_3_	90	90	90	38	48	0	0	0	0	0	0

**Table 15:** The total number of SMD types for each value of the λ parameter.

SMD	*λ*												
	0	0.1	0.2	0.3	0.4	0.5	0.6	0.7	0.8	0.9	1	min	max
*D*_1_	183	183	200	200	200	200	200	200	200	200	200	183	200
*D*_2_	97	97	97	97	125	125	125	125	125	125	100	97	125
*D*_3_	100	100	100	100	130	130	130	130	130	130	130	100	130
*D*_4_	132	132	132	132	132	132	145	145	145	145	145	132	145
*D*_5_	125	125	125	125	135	135	135	135	135	135	135	125	135

## Data Availability

Not applicable.
